# Validity and reliability of the 10-Item Adverse Childhood Experiences Questionnaire (ACE-10) among adolescents in the child welfare system

**DOI:** 10.3389/fpubh.2023.1258798

**Published:** 2023-11-17

**Authors:** Barnabás Oláh, Zita Fekete, Ildikó Kuritárné Szabó, Beáta Kovács-Tóth

**Affiliations:** ^1^Department of Behavioural Sciences, University of Debrecen Faculty of Medicine, Debrecen, Hungary; ^2^Doctoral School of Health Sciences, University of Debrecen, Debrecen, Hungary

**Keywords:** adolescence, adverse childhood experiences, child welfare system, family dysfunction, maltreatment, measures, psychometrics, validation

## Abstract

**Introduction:**

Multiple evidence suggests that the vast majority of children in the Child Welfare System (CWS) are victims of early, chronic, and multiple adverse childhood experiences. However, the 10-item version of the Adverse Childhood Experiences Questionnaire (ACE-10) has never been tested in such a particularly vulnerable population as adolescents living in the CWS. We aimed to assess the psychometric properties of the ACE-10 in a community sample of 240 Hungarian adolescents placed in family style group care (FGC) setting.

**Methods:**

Demographic data, the 10-item version of the Adverse Childhood Experiences Questionnaire (ACE-10), the Strengths and Difficulties Questionnaire (SDQ), and the HBSC Bullying Measure were used.

**Results:**

Our results showed acceptable internal consistency (α = 0.701) and item-total correlations (*r*_pb_ = 0.25–0.65, *p* < 0.001). However, our results also reflect that item 6 (“Parental separation/divorce”) is weakly correlated with both the cumulative ACE score and the rest of the questionnaire items. When item 6 is removed, the 9-item version of the ACE produces more favorable consistency results (α = 0.729). Strong and significant associations of the cumulative ACE score with emotional and behavioral symptoms and bully victimization confirm the concurrent criterion validity of both versions of the instrument.

**Discussion:**

Our findings suggest that ACE-9 and ACE-10 are viable screening tools for adverse childhood experiences in the CWS contributing to the advancement of trauma-informed care. We recommend considering the use of either the 9-item or the 10- item version in the light of the characteristics of the surveyed population. The implications and limitations are discussed.

## 1 Introduction

### 1.1 Child maltreatment and its consequences

The social determinants of health are the conditions in which people are born, grow, work, live, and age, and the broader set of forces and systems shaping the conditions of daily life (1). The social determinants of health represent a broader concept, whereas child maltreatment covers its specific aspect related to the family context. Poverty, unemployment, low socio-economic status and resulting chronic stress, and family structure characteristics (e.g., divorce, household substance use, household mental illness) are risk factors for child maltreatment (2, 3). The ecological model of the etiology of child abuse views maltreatment as a system of interacting risk and protective factors at four levels: the individual or ontogenic level, the family microsystem, the contextual level, and the social macrosystem (4). Accordingly, child maltreatment contributes to maintaining unfavorable social positions.

Child maltreatment (neglect, emotional, physical, sexual abuse) is a major public health issue worldwide. Over the past 20 years, studies were launched to explore child maltreatment on an increasingly broad scale. Some studies attempted to explore the prevalence of different forms of child abuse and neglect (5–10), whereas others focused on exploring their risk factors (2, 3, 11–13) and consequences (14–16). Recent research, however, has started examining a wider range of adverse factors affecting children; therefore, instead of using the term “child maltreatment,” we will use the broader term extended “adverse childhood experiences” (ACEs), which also includes dysfunctional family environments (household dysfunction) in addition to maltreatment (neglect and abuse) (15). Research has confirmed that adverse childhood experiences within the family are strong predictors of mental disorders and somatic outcomes, including chronic diseases (14–16).

### 1.2 Children in the child welfare system

Children in child protection are one of the most vulnerable populations, supporting their growth and providing them with psychological/social care is a major challenge worldwide (17). Children who are displaced from their biological families have a much more difficult life path (18). Children in CWS have a high prevalence of various adverse childhood experiences, ranging from 28 to 80% (19–26). The different prevalence results of these studies are influenced by differences in definitions and groupings of adversities/trauma, differences in measurement instruments, as well as age differences in the sample (27).

The conceptual model ACE pyramid represents the life-long consequences of persistent adverse experiences in childhood (28). Early onset, cumulative and prolonged adverse experiences can result in disrupted neurodevelopment, immune and endocrine system modifications (29–31). Attachment difficulties, emotional and behavioral dysregulation result in a lack of appropriate problem-solving (or coping mechanism) strategies, leading some of those exposed to adversity to engage in persistent health-damaging behaviors (28, 32–34). Some of these children suffering from social, emotional and cognitive impairments will develop mental and somatic illnesses over their life course, which leads to disability and/or social impairments later in life (28, 31).

### 1.3 Measuring adverse childhood experiences

Understanding the prevalence and characteristics of adverse childhood experiences is essential for planning interventions aimed at reducing child maltreatment, this requires easy-to-use, reliable measurement tools (27, 35, 36).

ACEs can be measured through self-assessment questionnaires or expert interviews. The advantage of questionnaires is that they are economical, easy to administer and score, and ensure anonymity, which can reduce the chance of biased responses triggered by shame associated with trauma (35, 37). Self-report by adolescents is still found to be more reliable than agency records, parental reports of adolescent victimization or adult retrospective self-report (37, 38).

Measures used to assess the prevalence of adverse childhood experiences should use consistent concepts about the types of child maltreatment and the same stands for their definitions (39). To avoid underestimation, items in measures should be behavior-specific, not ambiguous or non-specific (40). Ideally, all five forms of child maltreatment (physical abuse, sexual abuse, emotional abuse, neglect, and household dysfunction) should be measured simultaneously, as a number of children experience resulting polyvictimization and its increased consequences (16, 41, 42).

In a previous study methods to assess ACEs among children and families were identified and compared (35). Measuring tools commonly used in children and adolescents (without attempting to be comprehensive): Yale-Vermont Adversity in Childhood Scale; Adverse Childhood Experiences Questionnaire; Adverse Childhood Experiences Abuse Short Form (ACE-ASF); Philadelphia Childhood Adversity Questionnaire (CAQ); Childhood Trauma Questionnaire-Short Form (CTQ-SF); Juvenile Victimization Questionnaire (JVQ) (35, 43–45).

The 10-item ACE Score Calculator examines simultaneously all five forms of child maltreatment outlined above, with clear, unambiguous questions that are behavior-specific and concrete. In this way, the questionnaire is also suitable for testing an adolescent population in the child welfare system, so the clarity of the questions allows for the examination of children. To the best of our knowledge, only one study so far has examined the psychometric properties of this questionnaire in a normal adolescents population (46), although it is perfectly suitable for screening a larger population for adverse childhood experiences (as it can be applied simply and quickly).

The aim of our study was to assess the psychometric properties of the 10-item ACE Score Calculator and to demonstrate its reliability and validity in a specific sample of Hungarian adolescents placed in the Child Welfare System. There are no validated tools in Hungary for screening the vulnerable group of children living in CWS. The advances of the ACE-10 questionnaire mentioned above make it suitable for screening children in child welfare system for adverse childhood experiences. Increased attention to screening and assisting this population is needed, not only for the benefit of the population concerned, but also for the society as a whole.

## 2 Materials and methods

### 2.1 Sampling and data collection

The data collection on adolescents (aged 12˗17) under child protection services was carried out in 31 family-style group care (FGC) settings in 3 counties of Hungary, and was conducted between March 2018 and January 2020. The sampling frame for the CWS sample consisted of 309 mentally sound adolescents living in 31 FGCs. After having been provided oral and written information, the adolescents and their guardians signed the informed consent form to participate in the study. Adolescents filled in the questionnaire anonymously. The questionnaires were filled in during group sessions, where each three adolescents were supervised by one health psychology Msc student, whose help was mainly needed in case of reading or attention difficulties. When emotional, cognitive, or other reasons made it necessary, the questionnaires were completed in individual sessions instead. A total of 271 adolescents completed the questionnaire. The reasons for the missing responses were that some adolescents were not at home on the day of data collection, were runaways, or either the guardians or the adolescents refused to provide consent. After data cleaning (elimination of incompletely filled questionnaires), the final sample size was 240. Ethics approval was issued by the Research Ethics Committee of the Hungarian Medical Research Council under the approval number ETT TUKEB 47848-7/2018/EKU.

### 2.2 Measures

The *demographic data* of the studied adolescents were assessed using a self-developed questionnaire with items asking for information on gender, age, nationality, school type, grade, and residential location. We administered the Strengths and Difficulties Questionnaire (SDQ) (47, 48) and the HBSC (*Health Behavior in School-aged Children Questionnaire*) Bullying Measure (49, 50) to assess concurrent criterion validity of the ACE Score Calculator. Multiple studies have demonstrated that the social, emotional, and behavioral symptoms and roles in bullying (perpetration/victimization) are significant correlates of the accumulation of childhood adversities (16, 51–55).

ACEs were assessed using 10-item version of the Adverse Childhood Experiences Questionnaire (ACE-10), which is a retrospective self-report questionnaire consisting of 10 items (56). The questions in this survey aim to assess 10 types of early ACEs suffered before the age of 18. These ACEs cover the possible forms of maltreatment (physical, emotional, and sexual abuse, and physical, emotional neglect) and household dysfunction (parental separation/divorce, household physical violence, household substance abuse, household mental illness or suicide attempt, incarcerated household member). Previous research has demonstrated the different and distinct nature of these events through a series of analyses. This is why the questionnaire includes these items (15, 56, 57).

A cumulative ACE score between 0 and 10 is calculated by summing the number of ‘yes’ responses for each question, based on the number of types of ACEs. The ACE score is a severity index that measures the accumulation of different types of adverse experiences, showing how many types of adversities a person has experienced in their childhood. Our previous study confirms that the ACE-10 is suitable for assessing intrafamilial adverse childhood experiences in adolescents (46). The content of the items and the response options are provided in the Supplementary appendix.

For the assessment of social, emotional, and behavioral symptoms the *Strengths and Difficulties Questionnaire (SDQ)* was employed (47, 48). The items of this questionnaire can be grouped in five factors as follows: hyperactivity, emotional problems, behavioral disorders, peer relationship problems, and prosocial conduct. The Hungarian version of the Strengths and Difficulties Questionnaire was adapted and validated by Birkás et al. (58). The questionnaire was found to have acceptable internal consistency in the sample (Cronbach’s alpha = 0.70).

Bullying was assessed using the relevant questions of the *Health Behavior in School-Aged Children Questionnaire (HBSC-2014)* (49, 50). The HBSC questionnaire is a comprehensive measure of health behaviors administered among school-aged children every 4 years. The HBSC study is based on a standardized methodology and conducted in more than 40 countries in international collaboration with the World Health Organization (59). The HBSC Bullying Measure includes questions on bullying, the role of perpetrators and victims of physical and emotional abuse and cyberbullying, and participants of physical fight. Previous studies have reported good reliability of scales (Cronbach’s alpha = 0.76–0.84) (60, 61). First, the phenomenon of bullying was introduced, followed by four questions about bullying (in-person bullying victimization, cyberbullying victimization, bullying perpetration, and physical fight). In all the four categories the answers to choose from were: never/once or twice/2 or 3 times a month/about once a week/ several times a week. After combining the categories of bullying variables, we defined frequency as a binary variable (never vs. at least once in the past 12 months) (61).

### 2.3 Statistical analysis

Statistical analyses were carried out using IBM SPSS Statistics ver. 23.0 (IBM, Armonk, NY, United States). The sociodemographic and ACE characteristics, the mean and standard deviation of SEB symptom scores, and the frequency of bullying variables were described in the sample overall and by gender. Psychometric properties of the ACE Score Calculator were investigated through internal consistency (Cronbach’s alpha calculation), intercorrelations (Phi correlation), item-total correlations (Point-biserial correlations), and association analyses for concurrent criterion validity. Since the studies conducted during the development and evolution of the test confirmed the distinct nature of the events tested in each item, the dimensionality of the test was not examined (15, 56, 57).

Generalized linear models with entry method were used to test associations of ACE score with SEB symptoms, and logistic regression models with backward (Wald) method for ACE score with bullying variables. All models were adjusted for age, gender, and location, and post-test analysis was carried out with the help of the adjusted Wald test.

## 3 Results

### 3.1 Descriptive statistics of the sample

The total sample consisted of 240 adolescents [54.17% girls, mean age 14.9 (SD = 1.58)]. 7.5% (*n* = 17) of them reported no ACE, while 22.4% (*n* = 51) reported one, 18% (*n* = 41) reported two, 11.8% (*n* = 27) reported three and 40.4% (*n* = 92) reported four or more ACEs.

The most frequent type of reported child maltreatment was emotional abuse in 32.1% (*n* = 76), and emotional neglect in 30.1% (*n* = 71) of the sample. The least prevalent reported child maltreatment was sexual abuse in 13.6% (*n* = 32) of the respondents. Parental divorce or separation was reported by 71.2% of the adolescents (*n* = 196), which was followed by incarcerated household member 48.5% (*n* = 114) and household substance abuse (32.5%, *n* = 76). These three dysfunctions were the most prevalent reported dysfunctional household conditions, while the least prevalent was having experienced household physical violence (21.5%, *n* = 51) (Table 1).

**Table 1:** Demographic and ACE characteristics of the sample, overall and by gender.

	Boys *n* = 110 (45.8%)	Girls *n* = 130 (54.2%)	Total sample *n* = 240
Age mean (SD)	14.59 (1.59)	15.15 (1.53)	14.9 (1.58)
**Location *n* (%)**			
Village	19 (17.3)	10 (7.7)	29 (12.1)
Town	64 (58.2)	91 (70.0)	155 (64.6)
City	27 (24.5)	29 (22.3)	56 (23.3)
ACE score mean (SD)	3.1 (2.17)	3.2 (2.36)	3.16 (2.27)
**ACE score *n* (%)** ^a^			
0	8 (7.5)	9 (7.4)	17 (7.5)
1	21 (19.8)	30 (24.6)	51 (22.4)
2	24 (22.6)	17 (13.9)	41 (18.0)
3	13 (12.3)	14 (11.5)	27 (11.8)
4	7 (6.6)	18 (14.8)	25 (11)
5	19 (17.9)	16 (13.1)	35 (15.4)
6	4 (3.8)	8 (6.6)	12 (5.3)
7	6 (5.7)	2 (1.6)	8 (3.5)
8	4 (3.8)	1 (0.8)	5 (2.2)
9	–	7 (5.7)	7 (3.1)
10	–	–	–
Emotional abuse	32 (29.6)	44 (34.1)	76 (32.1)
Physical abuse	28 (25.7)	32 (24.8)	60 (25.2)
Sexual abuse	17 (15.7)	15 (11.8)	32 (13.6)
Emotional neglect	30 (27.8)	41 (32)	71 (30.1)
Physical neglect	20 (18.3)	19 (14.7)	39 (16.4)
Parental separation/divorce	74 (67.9)	94 (74)	168 (71.2)
Household physical violence	21 (19.3)	30 (23.8)	51 (21.7)
Household substance abuse	33 (30.3)	43 (34.4)	76 (32.5)
Household mental illness	23 (21.1)	34 (26.8)	57 (24.2)
Incarcerated household member	57 (52.3)	57 (45.2)	114 (48.5)

a The category does not add up to the full sample size due to some missing data.

Table 2 presents the descriptive statistics of SEB symptoms and bullying variables in the sample. The mean scores of SEB symptoms ranged from 3.00 (SD = 1.60) (conduct problems) to 3.64 (SD = 1.80) (hyperactivity/inattention problems), while the mean for the total difficulties score was 12.81 (SD = 5.43).

**Table 2:** Social, emotional, and behavioral (SEB) symptoms and bullying characteristics of the sample overall and by gender.

	Boys *n* = 110 (45.8%)	Girls *n* = 130 (54.3%)	Total sample *n* = 240
**SEB symptoms mean (SD)**			
Emotional symptoms	2.66 (2.34)	3.42 (2.61)	3.08 (2.52)
Conduct problems	2.94 (1.65)	3.05 (1.57)	3.00 (1.60)
Hyperactivity/inattention problems	3.63 (1.97)	3.66 (1.65)	3.64 (1.80)
Peer relationship problems	3.09 (1.98)	3.18 (1.79)	3.14 (1.87)
Total difficulties	12.17 (5.77)	13.32 (5.11)	12.81 (5.43)
**Bullying variables *n* (%)**			
Any type of victimization	36 (36.7)	69 (57.0)	105 (47.9)
In-person bullying victimization (in FGC)	21 (19.1)	44 (34.6)	65 (27.4)
In-person bullying victimization (in school)	14 (13.2)	28 (22.2)	42 (18.1)
Cyberbullying victimization	27 (26.7)	47 (37.0)	74 (32.5)
Bullying perpetration	23 (21.3)	35 (28.2)	58 (25.0)
Physical fight	65 (60.2)	76 (58.5)	141 (59.2)

In terms of bullying, almost half of the respondents (47.9%, *n* = 105) reported some type of victimization. The most prevalent form of bullying was physical fight (59.2%, *n* = 141) and cyberbullying victimization (32.5%, *n* = 74).

Around one-quarter reported in-person bullying victimization (27.4%, *n* = 65) and bullying perpetration (25%, *n* = 58).

### 3.2 Internal consistency and intercorrelations of the 10-item version of the ACE score calculator

The Cronbach’s alpha reliability shows an acceptable internal consistency (α = 0.700).

Intercorrelations of ACEs (Table 3) were used to study the strength of associations between the frequency of occurrence of each adverse event. The item “Parental separation/divorce” barely correlated with the other types of ACEs, and the item “Sexual abuse” only correlated with four of the rest of the adverse events.

**Table 3:** Intercorrelations between adverse childhood experiences (ACEs).

	Emotional abuse	Physical abuse	Sexual abuse	Emotional neglect	Physical neglect	Parental separation/divorce	Household physical violence	Household substance abuse	Household mental illness	Incarcerated household member
Emotional abuse	–									
Physical abuse	0.48^***^	–								
Sexual abuse	0.17^*^	0.10	–							
Emotional neglect	0.23^***^	0.27^***^	0.10	–						
Physical neglect	0.22^***^	0.33^***^	0.14^*^	0.19^**^	–					
Parental separation/divorce	−0.01	0.04	−0.02	−0.14	−0.09	–				
Household physical violence	0.29^***^	0.22^****^	0.26^***^	0.20^**^	0.32^***^	0.00	–			
Household substance abuse	0.17^*^	0.33^***^	0.13	0.11	0.21^***^	0.10	0.26^***^	–		
Household mental illness	0.19^**^	0.25^***^	0.17^*^	0.12^*^	0.20^**^	−0.04	0.30^***^	0.22^**^	–	
Incarcerated household member	0.19^***^	0.15^**^	0.16^**^	0.08	0.33^***^	0.14^*^	0.14^*^	0.20^**^	0.21^**^	–

Phi correlation; ^*^p < 0.05; ^**^p < 0.01; ^***^p < 0.001.

### 3.3 Item-total correlation

Point-biserial correlations were carried out to investigate the item-total correlation of the questionnaire. Table 4 shows that “Parental separation/divorce” was the only item to show weak correlation with the cumulative ACE score. In the case of the rest of the items, at least moderate correlations were found, which suggests the appropriate item-total correlations of the scale. “Physical abuse” exhibited the strongest correlation with the cumulative ACE score.

**Table 4:** Item-total correlations of the ACE questionnaire on the studied adolescent sample.

	ACE cumulative score^a^
Emotional abuse	0.58^***^
Physical abuse	0.62^***^
Sexual abuse	0.41^***^
Emotional neglect	0.44^***^
Physical neglect	0.50^***^
Parental separation/divorce	0.21^***^
Household physical violence	0.54^***^
Household substance abuse	0.56^***^
Household mental illness	0.49^***^
Incarcerated household member	0.50^***^

a Point-biserial correlation coefficients; ***p < 0.001.

Reviewing the data on intercorrelations and item-total correlations, the item “Parental separation/divorce” proved to be the least correlating item with the rest of the test items and the cumulative score. Consequently, we decided to examine the concurrent criterion validity of the test when the ACE 6 item (“Parental separation/divorce”) is removed.

### 3.4 Concurrent criterion validity of the 9-item version of the ACE

Considering the above results, the concurrent criterion validity was tested for both the 10-item and the 9-item version (excluding the item for parental separation/divorce). The comparison yielded similar results in the two versions with equal or somewhat stronger predictive potentials in the 9-item version. We used generalized linear models with entry method to examine the associations between ACE accumulation and SEB symptoms adjusted for age, gender, and location. The cumulative ACE score was significantly associated with more emotional symptoms (ACE-10: *B* = 0.20, *p* = 0.005; ACE-9: *B* = 0.23, *p* = 0.002), conduct problems (ACE-10: *B* = 0.15 *p* = 0.001; ACE-9: *B* = 0.16; *p* = 0.001), hyperactivity/inattention symptoms (ACE-10: *B* = 0.16, *p* = 0.002; ACE-9: *B* = 0.18, *p* = 0.001), and overall difficulties (ACE-10: *B* = 0.50, *p* = 0.002; ACE-9: *B* = 0.57, *p* = 0.001). Peer relationship problems did not correlate with the cumulative ACE score in the sample (ACE-10: *B* = 0.05, *p* = 0.391; ACE-9: *B* = 0.07; *p* = 0.255).

Logistic regressions with backward (Wald) method were applied to examine the relationship between ACE and bullying variables. Models were adjusted for age, gender, and location. One point increase in the ACE score significantly increased the possibility of being a victim of any measured type of bullying by 12 and 20% in ACE-10 and ACE-9, respectively (ACE-10: OR = 1.12, *p* = 0.007; ACE-9: OR = 1.20, *p* = 0.009). In details, the correlation of ACE score with in-person bullying victimization in FGC was marginally significant in ACE-10 and significant in ACE-9 (ACE-10: OR = 1.14, *p* = 0.059; ACE-9: OR = 1.15, *p* = 0.44), while in-person victimization in school was not significant (ACE-10: OR = 1.13, *p* = 0.115; ACE-9: OR = 1.13, *p* = 0.139). At the same time, being a victim of cyberbullying was in significant positive relationship with ACE accumulation (ACE-10: OR = 1.17, *p* = 0.014; ACE-9: OR = 1.17, *p* = 0.022), which was significantly associated with increased odds of involvement in physical fight (ACE-10: OR = 1.27, *p* = 0.001; ACE-9: OR = 0.127, p = 0.001). The association between ACE score and bullying perpetration were not significant in the sample, but *p*-values were nearing the margin (ACE-10: OR = 1.12, *p* = 0.094; ACE-9: OR = 1.13, *p* = 0.086).

When calculating the Cronbach’s alpha reliability index of the 9-item version, we found a more favorable value than in the case of the 10-item version (α = 0.729).

## 4 Discussion

In the present study, we conducted the psychometric evaluation of the ACE-10 questionnaire in a sample of Hungarian adolescents in the CWS. We assessed the intercorrelations, item-total correlations, concurrent criterion validity, and reliability of the instrument. The 10-item short form of the original ACE questionnaire comprised questions measuring 5 types of maltreatment (physical, emotional, sexual abuse, physical, and emotional neglect), and 5 types of household dysfunction.

### 4.1 Reliability and validity

Consistently with our study on average adolescent population (46), the ACE-10 showed appropriate internal consistency and reliability with a Cronbach’s alpha of 0.70. For internal correlation, we found that sexual abuse is only correlated with dysfunctional family factors (apart from physical neglect). This may be explained by the fact that sexual abuse is the only item that refers not only to harm suffered within the family but also to abuse suffered outside the family (15). The CWS population is an at-risk population, where a severely dysfunctional family system increases the risk of exposure to harm outside the family. Accordingly, we can assume that the “yes” responses for these item do not only refer to events experienced within the family, as opposed to the rest of the items. The item “Parental separation/divorce” is only correlated with the item “incarcerated household member,” and the correlation there is only a weak one. The results found for these two items are not in line with the results of our study on the average population, where both “Sexual abuse” and “Parental separation/divorce” were correlated with the other items of the scale.

The ACE-10 scale assessed in the CWS population showed adequate item-total correlations. However, not all of the ACE-10 items were found to be equally relevant in the Point-biserial analysis. Again, it is the item “Patental separation/divorce” that barely correlated with the ACE cumulative score. This result differs from our study on the average sample, where this item showed a moderate correlation with the ACE score.

A possible explanation for the less favorable psychometric indicators of this item may be found in the wording of the item itself: “*Were your parents ever separated or divorced?*” This is meant to provide information on the fact of separation itself, but eventually raises other relevant questions. Did the parents ever live together? How old was the child when the parents separated? How old was the child when he/she was placed in CWS? When did the parents separate compared to the time of the child’s placement in CWS? Was this a high-conflict separation/divorce or not (62)? Was it the parents’ cohabitation or their separation that had a major contribution more on the child’s placement in CWS?

It is worth noting that 71% of the adolescents in the sample reported having separated parents. This means that the sample can be considered as almost homogeneous in this regard, which may also have contributed to the results described above.

In addition, it is important to note that Hungary has relatively high divorce rates (1.9–2.2/1,000 people) in the European Union (63). It is also known that divorce rates are higher among people of lower socio-economic status (52), which is the social segment from which the majority of children in CWS across Europe are reported (53). This raises the question whether parental separation/divorce as an adverse experience should be asked about in this population at all, and if the psychometric properties of the test were still adequate if the item was omitted.

Accordingly, in the next stage we removed the sixth item and checked the psychometric properties of the 9-item version of the ACE. Our results show that the 9-item version of the test yielded better psychometric properties on the CWS population. In the light of the results, we recommend the careful use of item 6 (“Parental separation/divorce”). We suggest that the relevance of this item should be considered after careful examination of the population to be studied. However, our results also indicate that both the 10-item version or the ACE 9-item version of ACE can provide reliable information with appropriate psychometric properties.

The concurrent criterion validity of the instrument was good in our sample. We found strong and cumulative associations of the total ACE score with emotional and behavioral symptoms and bully victimization. The prevalence and gender distribution of reporting bully perpetration and victimization were similar to those found in other studies, in which girls also dominated in both cases (64). However, contrary to our expectations, the severity of peer relationship problems was independent from the cumulative ACE score. Given the previous evidence on clear correlation between ACE accumulation and the severity of peer problems in the general adolescent population (55), the setting of the CWS may be a clear explanation for our findings. Price and Brew (65) conclude that displacement and transitions themselves present unique social challenges for children. These transitions force them to adapt to new social expectations, such as fitting in with a new peer group. When placed in structured settings like group care, their interactions with peers are limited. These can exacerbate social difficulties that are already present as a result of maltreatment. Taking all these into consideration, the problems of peer relationships are very much related to transitions and dependent on the social context, equally for those reporting fewer or more ACEs. ACEs are individual experiences, but the transition adolescents face and the challenges it brings are collectively present, and collectively make it difficult for these youngsters to build and maintain proper relationships.

### 4.2 Strengths and limitations

Our study respondents were adolescents, which was advantageous for our study: they are closer in time to the period when they experienced the adversities compared to adults. Although children and adolescents may generally differ from adults in their ability to understand long-term consequences and regulate behavior, their cognitive abilities are not significantly different from those of adults (66–68). Similarly, adolescents’ cognition and reliable episodic memory are sufficiently developed to allow their participation in this type of research (69, 70). The advantages of using scores to measure adverse childhood experiences lie in the simplicity of the scores, which facilitates their widespread application in policy, public health and clinical settings. It is particularly important to use an internationally accepted measure to focus attention on this population.

On the other hand, the disadvantage of using a cumulative score is that it fails to take into account the fact that there are other indicators of severity in addition to ACE cumulation, which may be particularly important when assessing children in child protection. The non-representativity of the sample allows no extrapolation of the results, and the sensitive nature of the topic may cause bias and can potentially reduce the willingness of reporting ACEs. Nevertheless, self-report by adolescents is still found to be more reliable than agency records, parental reports of adolescent victimization or adult retrospective self-report (37, 38). A psychometric limitation of our study is that in the absence of other validated trauma questionnaires in Hungarian, convergent validity could not be assessed. In addition, the reliability levels, although acceptable, are at the limit of acceptability. The number of items and sample size also suggest caution.

A further limitation to the results may be that adolescents living in CWS may not recognize the adversities as adversities, as they have been socialized in them. Furthermore, in their case, self-reporting may have a biasing effect as they may wish to be reunited with their family of origin. Also, dissociation and memory deficits may bias the results downwards (71).

## 5 Conclusion

The results indicate that both the 10-item version and the 9-item version (without item 6) of the ACE is a valid and reliable measure of childhood adversities among disadvantaged adolescents living in CWS. We suggest that the relevance of item 6 (“Parental separation/divorce”) should be considered in relation with the population under study. Screening for trauma among children in CWS and detailed investigation of trauma types is of utmost importance in CWS, as targeted interventions can be based on these data. In order to follow the principles of trauma-informed care (72, 73), a valid measurement tool is needed as a first step. The ACE questionnaire is a brief, time- and cost-efficient, easy-to-understand instrument, which makes it suitable for disadvantaged children, considering that early impairment can lead to reading, comprehension and learning difficulties (24, 31, 74). Its suitability for repeated testing is another advantage. We see it as an appropriate tool for the purposes of screening, research, and treatment planning.

## Data availability statement

The raw data supporting the conclusions of this article will be made available by the authors, without undue reservation.

## Ethics statement

The studies involving humans were approved by Ethics approval was issued by the Research Ethics Committee of the Hungarian Medical Research Council under the approval number ETT TUKEB 47848-7/2018/EKU. The studies were conducted in accordance with the local legislation and institutional requirements. Written informed consent for participation in this study was provided by the participants’ legal guardians/next of kin.

## Author contributions

BO: Writing – original draft, Writing – review & editing. ZF: Writing – original draft, Writing – review & editing. IKSZ: Writing – review & editing, Supervision. BK-T: Writing – review & editing, Writing – original draft.
