# Supplementary material for: Validity and reliability of the 10-Item Adverse Childhood Experiences Questionnaire (ACE-10) among adolescents in the child welfare system
Source: Front Public Health. 2023 Nov 17;11:1258798. doi: 10.3389/fpubh.2023.1258798 (PMC10691263; doi:10.3389/fpubh.2023.1258798)
Supplement: Supplementary file 1 [file Table_1.docx]

Appendix

**The ACE-10—preambles, item contents and response options**

| **Item** | **Preamble and Content** | **ACE Category** |
| --- | --- | --- |
|  | During your life: |  |
| 1^a^ | Did a parent or other adult in the household often or very often ... Swear at you, insult you, put you down, or humiliate you? or Act in a way that made you afraid that you might be physically hurt? | Emotional abuse |
| 2^a^ | Did a parent or other adult in the household often or very often ... Push, grab, slap, or throw something at you? or Ever hit you so hard that you had marks or were injured? | Physical abuse |
| 3^a^ | Did an adult person at least 5 years older than you ever ... Touch or fondle you or have you touch their body in a sexual way? or Attempt or actually have oral, anal, or vaginal intercourse with you? | Sexual abuse |
| 4^a^ | Did you often or very often feel that ... No one in your family loved you or thought you were important or special? or Your family didn’t look out for each other, feel close to each other, or support each other? | Emotional neglect |
| 5^a^ | Did you often or very often feel that ... You didn’t have enough to eat, had to wear dirty clothes, and had no one to protect you? or Your parents were too drunk or high to take care of you or take you to the doctor if you needed it? | Physical neglect |
| 6^a^ | Were your parents ever separated or divorced? | Parental separation/divorce |
| 7^a^ | Was your mother or stepmother: Often or very often pushed, grabbed, slapped, or had something thrown at her? or Sometimes, often, or very often kicked, bitten, hit with a ﬁst, or hit with something hard? or Ever repeatedly hit for at least a few minutes or threatened with a gun or knife? | Witnessing violent treatment of mother |
| 8^a^ | Did you live with anyone who was a problem drinker or alcoholic or who used street drugs? | Household substance abuse |
| 9^a^ | Was a household member depressed or mentally ill, or did a household member attempt suicide? | Household mental illness |
| 10^a^ | Did a household member go to prison? | Incarcerated household member |
|  | ^a^ Dichotomous scales–yes/no. |  |

**Ártalmas Gyermekkori Élmények Kérdőív (ACE-10)**

**Kérdőív- instrukció, itemek tartalma és válaszlehetőségek**

| **Item** | **Instrukció és az itemek tartalma** | **ACE kategóriák** |
| --- | --- | --- |
|  | A következő kérdések a gyermekkorodra vonatkoznak. Kérjük, hogy amennyiben az alábbi állítások bármelyikét átélted ebben az időszakban, jelöld meg! |  |
| 1^a^ | Valamelyik szülő vagy más a háztartásban élő felnőtt gyakran/ vagy nagyon gyakran: szidott, sértegetett, elnyomott, megalázott? vagy Olyan módon viselkedett, hogy attól féltél, hogy fizikailag megsérülsz? | Érzelmi abúzus |
| 2^a^ | Valamelyik szülő vagy más a háztartásban élő felnőtt gyakran/ vagy nagyon gyakran: meglökött, megütött, erősen megragadott, vagy valamit hozzád vágott? vagy Valaha is ütött meg olyan erővel, hogy annak látható nyoma maradt, és Te megsérültél? | Fizikai abúzus |
| 3^a^ | Egy Tőled legalább 5 évvel idősebb személy valaha: fogdosta vagy simogatta az intim testrészeidet, vagy Neked fogdosni/simogatni kellett a másik személy intim testrészeit? vagy Megpróbált vagy megvalósított Veled orális, vagy anális, vagy vaginális közösülést? | Szexuális abúzus |
| 4^a^ | Gyakran/ vagy nagyon gyakran érezted: hogy a családodból senki nem szeret, vagy nem tart Téged különlegesnek és fontosnak? vagy A családtagjaid nem figyeltek oda egymásra, nem érezték egymáshoz közel magukat, nem támogatták egymást? | Érzelmi elhanyagolás |
| 5^a^ | Gyakran/ vagy nagyon gyakran érezted, hogy: nem volt elég ennivalód, koszos ruhákat kellett viselned, és nem volt, aki megvédjen Téged? vagy A szüleid túlságosan sokat ittak vagy drogoztak, hogy gondodat viseljék, vagy orvoshoz vigyenek, amikor szükséged volt rá? | Fizikai elhanyagolás |
| 6^a^ | Elváltak vagy különéltek a szüled? | Szülők különélése/válása |
| 7^a^ | Az édesanyádat vagy nevelőanyádat: gyakran/ vagy nagyon gyakran meglökték, erősen megragadták, megütötték vagy valamit hozzávágtak? vagy Néha, gyakran, vagy nagyon gyakran rugdosták, harapták, ököllel vagy kemény tárggyal ütötték? vagy Valaha perceken át folyamatosan ütötték, vagy késsel, fegyverrel megfenyegették? | Anya ellen elkövetett erőszak szemtanúja |
| 8^a^ | Éltél együtt olyan személlyel, aki problémás ivó vagy alkoholista volt, vagy kábítószert használt? | Alkoholt vagy egyéb pszichoaktív szert túlzottan használó családtag |
| 9^a^ | Családodban küzdött valaki depresszióval, vagy szenvedett mentálisan betegségben, vagy kísérelt meg öngyilkosságot? | Mentális betegségben szenvedő, vagy öngyilkosságot megkísérelt családtag |
| 10^a^ | Előfordult a családodban, hogy valaki börtönbe került? | Börtönviselt családtag |
|  | ^a^Dichotóm skála – igen/nem válaszok adása lehetséges |  |
